# Vitrification Ability of Combined and Single Cryoprotective Agents

**DOI:** 10.3390/plants10112392

**Published:** 2021-11-06

**Authors:** Milos Faltus, Alois Bilavcik, Jiri Zamecnik

**Affiliations:** Crop Research Institute, Drnovska 507, 16106 Prague, Czech Republic; bilavcik@vurv.cz (A.B.); zamecnik@vurv.cz (J.Z.)

**Keywords:** cryopreservation, cryoprotectant, differential scanning calorimetry, glass transition, vitrification, water crystallization

## Abstract

Cryoprotective agents (CPA) are an important part of many current vitrification methods. The vitrification ability of CPAs influences the probability of the glass transition and water crystallization occurrence. Thermal characteristics and the vitrification ability of two combined CPAs (PVS2 and PVS3), common plant vitrification solutions, and four single CPAs (ethylene glycol, DMSO, glycerol, and sucrose), the components of the mentioned PVSs, were evaluated utilizing a differential scanning calorimetry (DSC) during standard cooling/warming rates of 10 °C min^−1^. The effect of solute concentration on their vitrification ability was shown in the CPAs tested. Four typical concentration regions at which the glassy state and/or crystallization occurred were defined. We suggest the solute concentration of 0.7 g g^−1^ as the universal vitrification concentration, characterized by an actual Tg of CPA solution and limited water crystallization. Knowledge of the thermal properties of CPAs allows the design of new combined CPAs with the required vitrification ability respecting the cryopreservation method used and the characteristics of the cryopreserved sample.

## 1. Introduction

Cryopreserved organisms or their parts can be kept unchanged for many years. The reason for their stability is not only the low temperature itself, which slows down all processes or biochemical reactions but the specific state of matter [1]. During the cryopreservation process, the liquid state of the matter changes to a solid, so-called glassy state, and these conditions are maintained throughout the cryopreservation [2]. The solid-state of the matter is a source of stability in the cryopreserved material, due to its extreme viscosity [3]. There are no significant changes in the solid-state of the matter. The glassy state preserves the original structure of matter, in contrast to the growth of ice crystals [4]. Changes associated with the crystal arrangement of matter result in damage to the original structure and function and caused serious damage to living organisms [5]. The phase transition from the liquid to the glassy state of matter is called the glass transition and this process is called vitrification [6].

Dehydration and/or vitrification of a cryopreserved living organism is a prerequisite for successful cryopreservation and stability of cryopreserved organisms [7]. All cryoprotocols (Table 1), which lead to the dehydration or vitrification of living organisms, reduce the water content to prevent the occurrence of harmful ice crystals during cooling to ultra-low temperatures [8]. These procedures may utilize natural acclimatization processes leading to a moderate reduction in water content and/or increase in tolerance to dehydration [9], but a procedure leading to a dramatic reduction in water content (Table 1) is always included [10]. This dehydration takes place by treatment with dry air over the silica gel or in a laminar flow-bench, the freeze dehydration during controlled slow cooling in a two-step cryopreservation method or by the action of osmotically active solutions, CryoProtective Agents (CPAs), which dehydrate living cells and help vitrify them during cooling [11]. The latter methods are usually called vitrification methods (Table 1) [12].

The CPAs can contain a mixture of non-penetrating and penetrating components [13]. The first acts outside the cells and osmotically dehydrates the protoplasts. The effect of penetrating cryoprotectants is combined. They act osmotically outside the cell and prevent the water crystallization after penetrating the cells, but usually show higher cytotoxicity compared to non-penetrating cryoprotectants [14].

CPA-based cryoprotocols are currently very popular [15,16,17], but most of them are developed more or less empirically by testing the effect of various CPA mixtures on cryopreserved organisms/organs/cells. In addition, cryoprotocols are unfortunately still not easily transferable to other laboratories [18] and this fact significantly limits their wider use.

The routine use of cryopreservation methods can be supported by the development of new cryoprotocols or modification of currently used ones based on exact knowledge of the characteristics of CPAs used. When developing a new CPA, several key points need to be addressed: (1) selection of a suitable method of dehydration (air, freeze, osmotic), (2) the type of liquid phase transition (freezing and/or vitrification), and (3) the CPA treatment (none/single/combined) [19,20]. The choice of these options (Table 1) depends on the properties of the cryopreserved material, in particular its ability to withstand at least mild dehydration naturally by air dehydration or freezing without CPA treatment, typical of some plant species [21]. If a cryopreserved object does not tolerate dehydration, usually animal or sensitive plant cells or tissues, the use of CPAs is necessary for the successful application of the cryopreservation method. CPAs can be used either to modify or to prevent water freezing [12]. In the first case, the CPA concentration is low and the (equilibrium) freezing of water is controlled at a slow cooling rate; in the latter case, the CPA concentration is high to skip water freezing at rapid cooling/warming (C/W) rates (Table 1). The Critical Cooling Rates (CCRs) and Critical Warming Rates (CWRs) have already been defined for some CPAs [22,23,24].

Based on the CPA concentration, the thermodynamically non-equilibrium vitrification approach can occur at three different conditions: (1) Unstable vitrification happen when solute concentration is very low and must be overcome by extremely high C/W rates, (2) metastable vitrification occurred at medium CPA concentration and moderate C/W rates, and (3) stable vitrification depends on very high CPA concentration and it is independent on the C/W rates [6,13]. The use of high concentrated CPAs is limited by their toxicity to living cells and therefore the appropriate concentration of CPAs must be validated [25]. With regard to the term stable vitrification, it should be noted that the glassy state itself is not a thermodynamically equilibrium or stable [6,26]. The glassy state is metastable because it is not in its lowest energy form, in contrast to the crystalline form of matter [26], which is thermodynamically favored [6]. In this context, the stable vitrification is not thermodynamically correct term but it rather indicates the tendency of the solution to vitrify [27].

The effectiveness of CPA concentration on successful cryopreservation depends on is ability to induce the glassy state without water crystallization, which is a function of the CPA concentration and the proportion and characteristics of the CPA components [13,16]. At least two types of glass transitions can be identified: (1) the actual glass transition of solution (Tg) and (2) the glass transition of maximally freeze-concentrated solution (Tg’), which is defined by a concentration (C_g’_), which occurred as a result of water freezing and progressive concentration of solution [28,29]. The Tg characterizes glass transition development without any water freezing but the Tg’ detection always shows ice crystals occurred during the cooling period [28,29,30]. Whereas the Tg indicates a reliable vitrification process, the Tg’ reveals the suboptimal vitrification conditions. Therefore, the development of new cryoprotocols can be improved through knowledge of the thermal characteristics of the CPAs, such as the melting/freezing point and the change in a specific heat capacity associated with matter phase transitions [31]. These parameters can be measured by differential scanning calorimetry [32,33,34,35,36,37,38,39]. The thermal characteristics of the cryoprotective solutions used and their components may help to select appropriate CPAs for specific cryopreservation methods concerning the cryopreserved material and the presence or avoidance of crystallization during C/W cycles [32,40].

This work solved the problem of selecting suitable CPAs for the development of reliable vitrification cryoprotocols, which are based on the ability of CPAs to induce a glassy state without harmful water crystallization, which we defined as the vitrification ability. This paper aimed to analyze the effect of solute concentration on thermal characteristics and the vitrification ability of two common multiple CPAs, the Plant Vitrification Solution 2 (PVS2) and the Plant Vitrification Solution 3 (PVS3), and four single CPAs: ethylene glycol (EG), dimethyl sulfoxide (DMSO), glycerol (Gly), and sucrose (Suc), which are the components of the combined CPAs tested.

## 2. Results

### 2.1. Vitrification Ability of PVS2-Based Solutions

**Figure 1:**
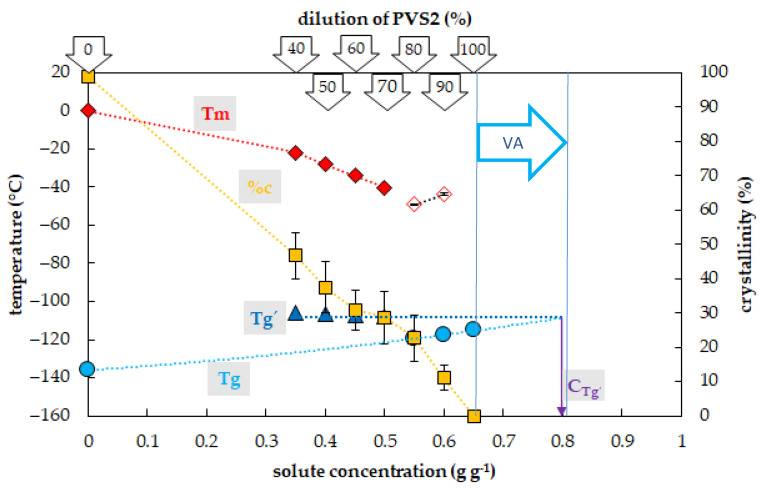
Thermal characteristics of osmotic components of the Plant Vitrification Solution 2 (PVS2). The *x*-axis expresses the total concentration of solutes (g) per mass of aqueous solution (g). The left *y*-axis indicates the temperature of thermal events, the right *y*-axis shows the percentage of water crystallinity on the total mass of solution. The temperature of the glass transition (Tg) of the solution is indicated by a cyan circle, the temperature of the glass transition Tg’ of the freeze-concentrated solution is indicated by a blue triangle, the onset of the water melting peak (Tm) is indicated by a red diamond if the effect occurs during the cooling period, the onset of the water melting peak is indicated by a hollow diamond if the effect occurs during the warming period. The percentage of water crystallinity (%c) based on the total mass of the solution is indicated by an orange square. The Cg’ value marks the concentration of the freeze-concentrated solution. The vitrification ability (VA) demonstrates the range of solute concentrations with the appropriate vitrification ability. All effects were measured during the warming period of C/W cycles at a rate of 10 °C min^−1^. Results are presented as a mean of three repetitions and vertical bars represent standard error.

The crystallization of water in the PVS2-based solutions during the cooling cycle occurred in a range of concentrations from 0.35 to 0.5 g g^−1^, which corresponded to 40–70% of the PVS2 concentration (Figure 1). In this region, the percentage of water crystallization declined from 47% to 29% of the total mass of the sample with increasing solute concentration. No water crystallization occurred during the cooling cycle in solutions with a higher concentration of solutes. Water crystallization during the warming cycle was detected in solutions with concentrations of solutes of 0.55 and 0.6 g g^−1^ (80% PVS2 and 90% PVS2, respectively). The curve of crystallinity rapidly dropped to 23 and 11% of the total mass of the sample within these solute concentrations. No water crystallization was detected in the most concentrated solution containing 0.65 g g^−1^ (100% PVS2) during C/W cycles.

The occurrence and the value of the glass transition temperature of PVS2-based solutions tested were influenced by a solute concentration and two curves of Tg were detected (Figure 1). The first, the curve of Tg’, was always accompanied by water crystallization during the cooling cycle and occurred at low solute concentrations (from 0.35 to 0.5 g g^−1^). At the intersection with the Tg curve, the Tg’ curve indicated a concentration (C_g’_) of the solution originating from the water freezing in the diluted solutions, which occurred near 0.8 g g^−1^ (Figure 1). The Tg’ was independent of original solute concentrations and it oscillated from −108 to −106 °C (Figure 1). The second, the curve of Tg, was detected at high solute concentrations (from 0.55 to 0.65 g g^−1^) when no water crystallization occurred during the cooling cycle. It was dependent on solutes concentration when it increased from −120 to −115 °C with increasing solute concentration (Figure 1).

### 2.2. Vitrification Ability of PVS3-Based Solutions

**Figure 2:**
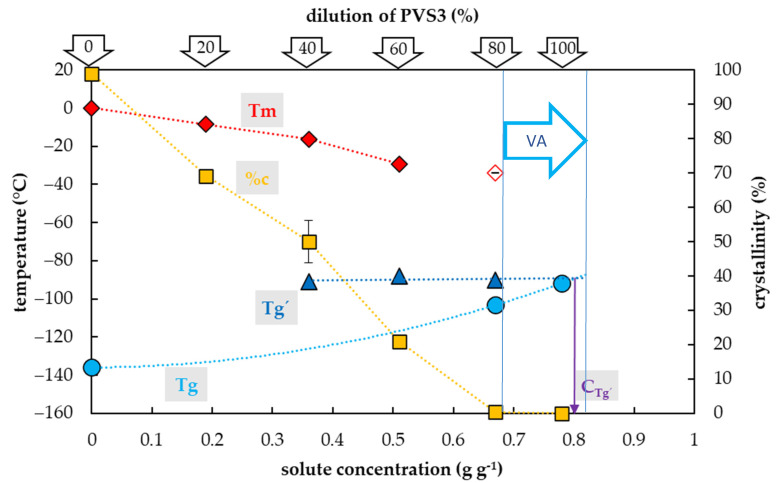
Thermal characteristics of osmotic components of the Plant Vitrification Solution 3 (PVS3) depending on their concentrations in aqueous solution. The total concentration of solutes is expressed as the mass of solutes (g) per mass of aqueous solution (g). The left *y*-axis indicates the temperature of thermal events, the right *y*-axis shows the percentage of water crystallinity based on the total mass of solution. The temperature of the glass transition (Tg) of the solution is indicated by a cyan circle, the Tg’ originating from freeze-concentrated solution during the cooling period is indicated by a blue triangle, the onset of the water melting peaks (Tm) is indicated by a red diamond if the effect occurs during the cooling period, the onset of the water melting peak is indicated by a hollow diamond if the effect occurs during the warming period. The percentage of water crystallinity (%c) based on the total mass of the solution is indicated by an orange square. The vitrification ability (VA) demonstrates the range of solute concentrations with the appropriate vitrification ability. Cg’ indicates a concentration of the freeze-concentrated solution. All effects were measured during the warming period of C/W cycles at a rate of 10 °C min^−1^. Results are presented as a mean of three repetitions and vertical bars represent standard error.

Crystallization of water in the PVS3-based solutions during the cooling cycle occurred in the range of concentrations from 0.19 to 0.5 g g^−1^, which corresponded to 20–60% of the PVS3 concentration (Figure 2). In this region, the percentage of water crystallization decreased from 69% to 21% of total sample mass with increasing solute concentration. No water crystallization occurred during the cooling cycle in solutions with higher concentrations of solutes. Negligible water crystallization during the warming cycle was detected in a solution with a solute concentration of 0.67 g g^−1^ (80% PVS3). At this solute concentration, the crystallinity curve dropped dramatically to 0.4% of the total sample mass. In the most concentrated solution (100% PVS3) containing 0.78 g g^−1^ of solutes, no water crystallization was detected during the C/W cycles.

The glass transition of PVS3-based solutions tested was also influenced by a solute concentration and the presence of water crystallization and two curves of glass transition temperatures were detected (Figure 2). The first, the curve of Tg’, was always accompanied by water crystallization during the cooling cycle and occurred at low solute concentrations (from 0.19 to 0.5 g g^−1^). It was independent of solute concentrations and oscillated from −90 to −88 °C (Figure 2). At the intersection with the Tg curve, the Tg’ curve indicated a concentration (C_g’_) of the solution originating from the water freezing in the diluted solutions, which occurred near 0.8 g g^−1^ (Figure 2). The second, the curve of Tg, was detected at high solute concentrations (from 0.67–0. 78 g g^−1^) when no water crystallization occurred during the cooling cycle. It was dependent on solutes concentration when increased from −103 to −92 °C with increasing solute concentration (Figure 2).

### 2.3. Vitrification Ability of the EG Solutions

**Figure 3:**
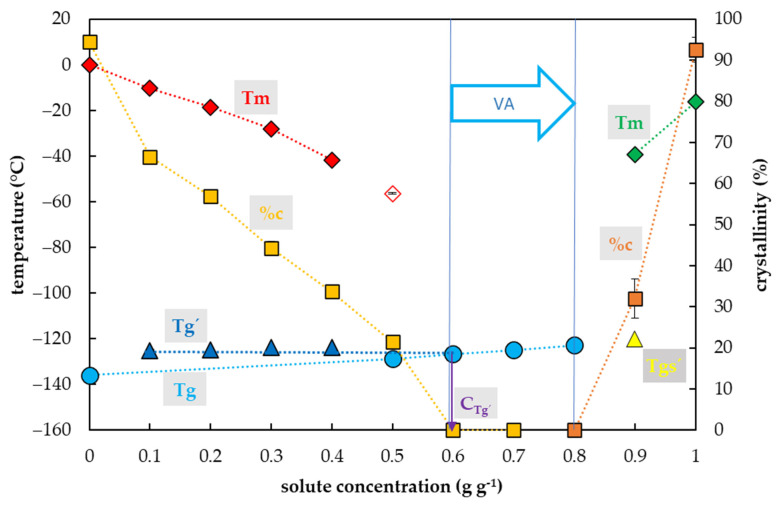
Thermal characteristics of ethylene glycol (EG) depending on its concentration in an aqueous solution. The total concentration of solutes is expressed as the mass of solutes (g) per mass of aqueous solution (g). The left *y*-axis indicates the temperature of the thermal events, the right *y*-axis shows the percentage of crystallinity of water or EG based on the total mass of the solution. The temperature of the glass transition (Tg) of the solution is indicated by a cyan circle, the Tg’ originating from the freeze-concentrated solution during the cooling period is indicated by a blue triangle, the Tgs’ originating from the EG crystallization is indicated by a yellow triangle, the onset of water melting peaks (Tm) is marked by a red diamond if the effect occurs during the cooling period and indicated by a hollow diamond if the effect occurs during the warming period. The onset of the EG melting peaks (Tm) is indicated by a green diamond. The percentage of crystallinity of the solvent or solute (%c) based on the total mass of the solution is indicated by an orange square for water and a brown square for EG. The vitrification ability (VA) demonstrates the range of solute concentrations with the appropriate vitrification ability. Cg’ indicates a concentration of the freeze-concentrated solution. All effects were measured during the warming period of C/W cycles at a rate of 10 °C min^−1^. Results are presented as a mean of three repetitions and vertical bars represent standard error.

Crystallization of water in the EG solutions during the cooling cycle occurred in a range of EG concentrations from 0.1 to 0.4 g g^−1^ (Figure 3). In this region, the percentage of water crystallization decreased from 67% to 34% of a total sample mass with the increasing EG concentration. No water crystallization occurred during the cooling cycle in solutions with a higher EG concentration. Crystallization of water during the warming cycle was detected in a solution with the EG concentration of 0.5 g g^−1^. 

The glass transition of the EG solutions tested was influenced by EG concentrations and two curves of glass transition temperatures were detected (Figure 3) as in the case of combined CPAs. The first, the curve of Tg’, was always accompanied by water crystallization during the cooling cycle and occurred at low EG concentrations (from 0.1 to 0.4 g g^−1^). It was independent of solute concentrations and oscillated from −125 to −124 °C (Figure 3). At the intersection with the Tg curve, the Tg’ curve indicated a concentration (C_g’_) of the solution originating from the water freezing in the diluted solutions, which occurred near 0.7 g g^−1^ (Figure 3). The second, the curve of Tg, was detected at high solute concentrations (from 0.5–0.9 g g^−1^) when no water crystallization occurred during the cooling cycle. It was dependent on solutes concentration; it increased from −129 to −120 °C (Figure 3).

### 2.4. Vitrification Ability of the DMSO Solutions

**Figure 4:**
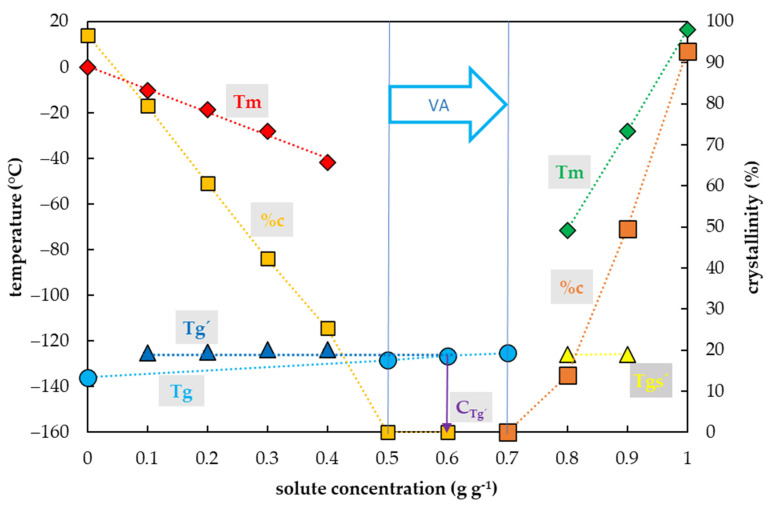
Thermal characteristics of dimethyl sulfoxide (DMSO) depending on its concentration in an aqueous solution. The total concentration of solutes is expressed as the mass of solutes (g) per mass of aqueous solution (g). The left *y*-axis indicates a temperature of thermal events, the right *y*-axis shows the percentage of crystallinity of water or DMSO based on the total mass of the solution. The temperature of the glass transition (Tg) of the solution is indicated by a cyan circle, the Tg’ originating from the freeze-concentrated solution during the cooling period is indicated by a blue triangle, the Tgs’ originating from the DMSO crystallization is indicated by a yellow triangle, the onset of water melting peaks (Tm) is indicated by a red diamond if the effect occurs during the cooling period, the onset of the DMSO melting peaks (Tm) is indicated by a green diamond. The percentage of crystallinity of the solvent or solute (%c) based on the total mass of the solution, is indicated by an orange square for water and a brown square for DMSO. The vitrification ability (VA) demonstrates the range of solute concentrations with the appropriate vitrification ability. Cg’ indicates a concentration of the freeze-concentrated solution. All effects were measured during warming period of the C/W cycles at a rate of 10 °C min^−1^. Results are presented as a mean of three repetitions and vertical bars represent standard error.

Crystallization of water in the DMSO solutions during the cooling cycle occurred in a range of DMSO concentrations from 0.1 to 0.4 g g^−1^ (Figure 4). In this region, the percentage of water crystallization decreased from 80% to 25% of the total sample mass with an increasing DMSO concentration. No water crystallization occurred during the cooling cycle in solutions with higher concentrations of DMSO. No water crystallization was detected in solutions with 0.5 g g^−1^ or higher during the C/W cycles. However, a crystallization of DMSO was detected at concentrations of DMSO from 0.8 to 1.0 g g^−1^, and a portion of the crystallinity ranged from 14 to 100% of DMSO.

The glass transition of DMSO solutions tested was influenced by a DMSO concentration, similarly to former CPAs. Three curves of glass transition were detected (Figure 4). The first, the curve of Tg’, was always accompanied by water crystallization during the cooling cycle and occurred at low DMSO concentrations (from 0.1 to 0.4 g g^−1^). It was independent of solute concentrations and ranged from −126.2 to −126.5 °C (Figure 4). At the intersection with the Tg curve, the Tg’ curve indicated a concentration (C_g’_) of the solution originating from the water freezing in the diluted solutions, which occurred below 0.7 g g^−1^ (Figure 4). The second, the curve of Tg, was detected at high solute concentrations (from 0.5–0.7 g g^−1^) when no water crystallization occurred during the cooling cycle. It was dependent on solute concentrations; it increased from −128.4 to −125.3 °C. The third, the curve of Tgs’, was detected at the highest DMSO concentrations (from 0.8–0.9 g g^−1^) when the DMSO crystallization occurred during the cooling cycle. It was independent of solutes concentrations; it oscillated from −125.9 to −125.8 °C (Figure 4).

### 2.5. Vitrification Ability of the Gly Solutions

**Figure 5:**
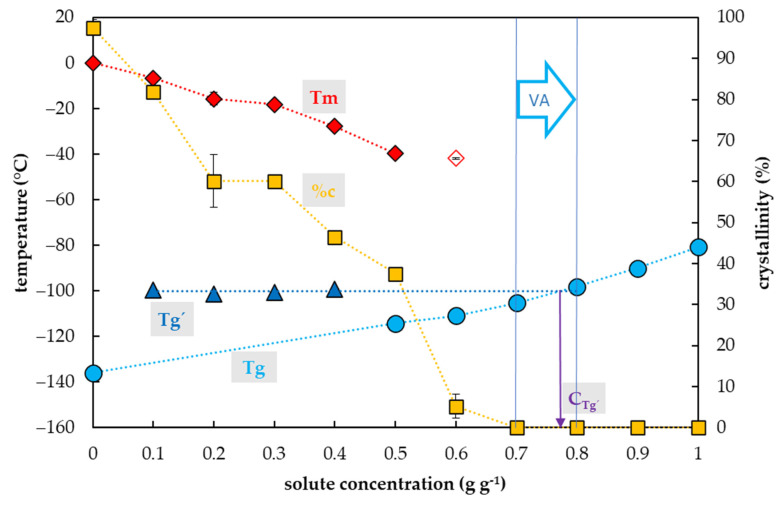
Thermal characteristics of glycerol (Gly) depending on its concentration in an aqueous solution. The total concentration of solutes is expressed as the mass of solutes (g) per mass of aqueous solution (g). The left *y*-axis indicates the temperature of thermal events and right *y*-axis shows the percentage of water crystallinity based on the total mass of solution. The temperature of the glass transition (Tg) of the solution is indicated by a cyan circle, the Tg’ originating from the freeze-concentrated solution during the cooling period is indicated by a blue triangle, onset of the water melting peaks (Tm) is indicated by a red diamond if the effect occurs during the cooling period, the onset of the water melting peak is indicated by a hollow diamond if the effect occurs during the warming period. The percentage of crystallinity of the solute (%c) based on the total mass of the solution is indicated by an orange rectangle. The vitrification ability (VA) demonstrates the range of solute concentrations with the appropriate vitrification ability. Cg’ indicates a concentration of the freeze-concentrated solution. All effects were measured during the warming period of the C/W cycles at a rate of 10 °C min^−1^. Results are presented as a mean of three repetitions and vertical bars represent standard error.

Water crystallization in the Gly solutions during the cooling cycle occurred in a range of concentrations from 0.1 to 0.5 g g^−1^ (Figure 5). In this region, the percentage of water crystallization decreased from 82% to 38% of the total mass of the sample with increasing Gly concentration. No water crystallization occurred during the cooling cycle in solutions with a higher concentration of Gly. Water crystallization was detected during the warming cycle in a solution with a Gly concentration of 0.6 g g^−1^. The curve of crystallinity dramatically dropped to 5% of the total mass of the sample in this Gly concentration. No water crystallization was detected in the Gly concentration of 0.7 g g^−1^ or higher during both the C/W cycles. 

The glass transition of Gly solutions was influenced by a Gly concentration and two curves of glass transition temperatures were detected (Figure 5). The first, the curve of Tg’, was always accompanied by water crystallization during the cooling cycle and occurred at low Gly concentrations (from 0.1 to 0.4 g g^−1^). It was independent of solute concentrations and oscillated from −101 to −99 °C (Figure 5). At the intersection with the Tg curve, the Tg’ curve indicated a concentration (C_g’_) of the solution originating from the water freezing in the diluted solutions, which occurred below 0.8 g g^−1^ (Figure 5). The second, the curve of Tg, was detected at high solute concentrations (from 0.5 to 1.0 g g^−1^) and was dependent on solute concentration when it was increased from −114 to −81 °C (Figure 5).

### 2.6. Vitrification Ability of the Suc Solutions

**Figure 6:**
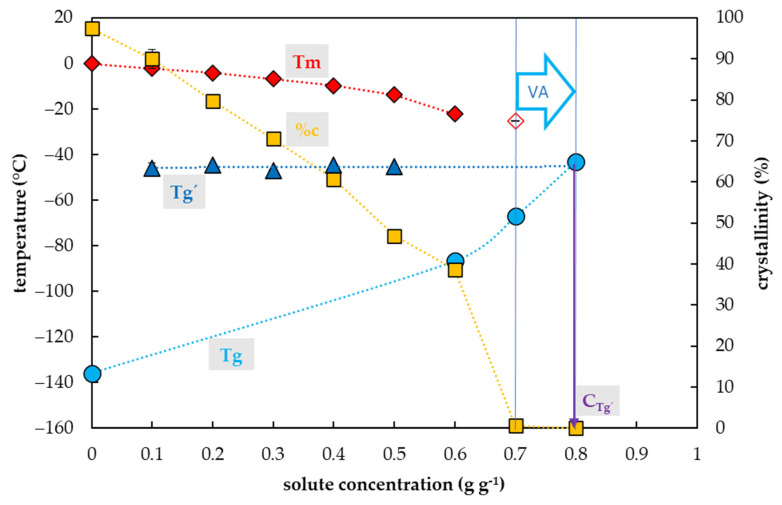
Thermal characteristics of sucrose (Suc) depending on its concentration in an aqueous solution. The total concentration of solutes is expressed as the mass of solutes (g) per mass of aqueous solution (g). The left *y*-axis indicates the temperature of thermal events, right *y*-axis shows the percentage of water crystallinity based on the total mass of the solution. The temperature of the glass transition (Tg) of the solution is indicated by a cyan circle, the Tg’ originating from the freeze-concentrated solution during the cooling period is indicated by a blue triangle, onset of the water melting peaks (Tm) is indicated by a red diamond if the effect occurs during the cooling period, the onset of the water melting peak is indicated by a hollow diamond if the effect occurs during the warming period. The percentage of crystallinity of water (%c) based on the total mass of the solution is indicated by an orange square. The vitrification ability (VA) demonstrates the range of solute concentrations with the appropriate vitrification ability. Cg’ indicates a concentration of the freeze-concentrated solution. All effects were measured during the warming period of the C/W cycles at a rate of 10 °C min^−1^. Results are presented as a mean of three repetitions and vertical bars represent standard error.

Water crystallization in the solutions during the cooling cycle occurred in a range of Suc concentrations from 0.1 to 0.6 g g^−1^ (Figure 6). In this region, the percentage of water crystallization decreased from 91% to 38% of the total mass of the sample with increasing Suc concentration. No water crystallization occurred during the cooling cycle in solutions with higher Suc concentrations. Negligible water crystallization was detected in a solution with the Suc concentration of 0.7 g g^−1^ (Figure 6). The curve of crystallinity dramatically dropped to 0.6% of the total mass of the sample at this Suc concentration. No water crystallization was detected in the most concentrated solution (0.8 g g^−1^ of Suc) during the C/W cycles.

The Tgs of Suc solutions tested were influenced by a Suc concentration and two curves of glass transition temperature were detected (Figure 6). The first, the curve of Tg’, was always accompanied by water crystallization during the cooling cycle and occurred at low Suc concentrations (from 0.1 to 0.5 g g^−1^). It was independent of solute concentrations and ranged from −47 to −45 °C (Figure 6). At the intersection with the Tg curve, the Tg’ curve indicated a concentration (C_g’_) of the solution originating from the water freezing in the diluted solutions, which occurred near 0.8 g g^−1^ (Figure 6). The second, the curve of Tg, was detected at high solute concentrations (from 0.6–0.8 g g^−1^) and was dependent on solutes concentration when increased from −87 to −43 °C (Figure 6).

## 3. Discussion

The thermal analysis of PVS2, PVS3, and their components demonstrated key features, the vitrification ability, and limits of these solutions for their use as CPAs in vitrification cryoprotocols concerning the CCR and CWR of 10 °C min^−1^.

### 3.1. PVS2-Based Solutions

The results showed that all diluted PVS2 solutions (40–90%) can be used as a step of graduate dehydration [3,41,42,43] as they decreased the amount of frozen water but they did not have satisfactory vitrification ability (Figure 1) at the C/W rates of 10 °C min^−1^. Only the most concentrated solution, the original (100%) PVS2 concentration, did not show any water crystallization in the C/W cycles (Figure 1) at the standard C/W rates. We proved that the PVS2 concentrations of 0.65 g g^−1^ or higher have an acceptable vitrification ability with no risk of water crystallization. We confirmed former findings that the CCR of PVS2 is less than 10 °C min^−1^ [44] but, moreover, we proved that the CWR of the PVS2 is less than 10 °C min^−1^ as well (Figure 1). A dilution of the PVS2 solution to 80–90% (0.55 and 0.6 g g^−1^) resulted in an overcoming ice crystallization during the cooling cycle but the crystallization still occurred during the warming cycle (Figure 1). We conclude that the CCR of 80% PVS2 is less than 10 °C min^−1^ in contrast to the CWR, which is higher than 10 °C min^−1^. The PVS2 solution diluted to 40–70% (0.35–0.5 g g^−1^) still showed the osmotic effect of the solution due to decreasing water crystallization but the vitrification ability was weak (Figure 1). We conclude that the water freezing at PVS2 concentrations below 0.55 g g^−1^ risks sample damage during the C/W cycles because the successful sample vitrification strongly depends on very high values of both the CCRs and the CWRs. 

Two types of Tg curves in the PVS2-based solutions were detected concerning the solute concentrations. The Tg’ curve was a result of water crystallization [13] during the cooling cycle in the concentration range of 40–70% of PVS2 solution but it did not correspond to the Tg value of the original solutions. The Tg’ value indicated the concentration of the freeze-concentrated solution (Cg’) (Figure 1). The Cg’ of the freeze-concentrated PVS2 solution was higher than the concentration of 100% PVS2 (Figure 1). The presence of Tg’ in the 40–70% PVS2 (Figure 1) indicated higher CCR than the actual cooling rate used [13]. The second curve of Tg was detected in a concentration range of 80–100% PVS2 (Figure 1) and represented the Tgs of the original solutions. It was also developed during the cooling cycle and ranged in temperatures from −120 to −115 °C (Figure 1). The DSC measurement confirmed a satisfactory vitrification ability of the 100% PVS2 solution at the standard C/W rates. The Tg of this solution at −115 °C corresponds to the value of the PVS2 solutions already published [38,45,46]. 

We proved that the detection of the actual Tg was not crucial for the solution vitrification ability because water crystallization still occurred in 80% and 90% PVS2 during warming cycles simultaneously with Tg presence (Figure 1). Insufficient cryoprotectant concentration or insufficient warming rate may result in uncontrolled crystallization of water during thawing of samples [44,47,48], even though the samples were safely stored at liquid nitrogen temperature without any damage. This problem can also occur even with an appropriate concentration of CPA, but for an insufficient treatment period to properly dehydrate the sample [3].

Only the 100% PVS2 concentration showed sufficient vitrification ability, characterized by the presence of a glassy state without any water crystallization at the C/W rates of 10 °C min^−1^. The CCR and CWR values of this solution are lower than 10 °C min^−1^, which does not place too high demands on the temperature course during vitrification. Diluted PVS2 can be used as an osmotic agent to gradually dehydrate a sample before its vitrification or as CPA at higher C/W rates than 10 °C min^−1^. This suggestion was successfully proved by the applicability of 80% PVS2 using the droplet-vitrification procedure [42]. The successful application of diluted PVS2 proved the higher C/W rates provided by the droplet-vitrification method, and thus is consistent with our results performed at the standard C/W rates of 10 °C min^−1^. Insufficient dehydration can result in water crystallization mostly during the warming or even cooling cycle, in the case that the CWR or CCR are not adequate for the sample solute concentration. The presence of the Tg’, which is associated with the freeze-concentrated solution, reveals an insufficient actual cooling rate, which is lower than the CCR of the solution, and simultaneously shows the solute concentration (Cg’) providing conditions (~0.8 g g^−1^) close to the stable vitrification conditions [6].

Two possible constraints of the CPA use should be taken into an account. A too extended period of dehydration can result in sample osmotic injury due to excessive water loss, and high concentration or presence of some penetrating components of CPAs can result in specific toxicity and injury of the cryopreserved material during vitrification [49,50]. To decrease the PVS2 toxicity the exposition at 0 °C is strongly recommended [46,51].

### 3.2. PVS3-Based Solutions

The results showed the vitrification ability of the PVS3-based solutions is satisfactory in a range from 80 to 10% of the original concentration (Figure 2). The most concentrated 100% PVS3 solution, did not show any water crystallization during the C/W cycles at the standard C/W rate. We can conclude that both the CCR and CWR are less than 10 °C min^−1^ in the 100% PVS3 solution. In the case of a solution diluted to 80% PVS3, no water crystallization occurred during cooling and only negligible water crystallization (0.4% *w/w*) was found during the warming cycle (Figure 2). This amount of frozen water was very close to the commonly agreed threshold value for the CCR at 0.2% of ice (*w/w*) [52] and the quantity of 0.5% *w/w* is not considered to be sufficient to cause any damage [52]. Moreover, real C/W rates are usually higher than 10 °C min^−1^ in the vitrification methods. Therefore, we consider 80–100% PVS3 solutions as appropriate CPAs with reliable vitrification ability for most vitrification methods. Our suggestions are in an agreement with the finding that the PVS3 solution in a concentration range from 80 to 100% resulted in acceptable cell survival (77.1–82.6%) [53]. More diluted PVS3 solutions (20%–60%) showed water crystallization during the cooling cycle (Figure 2) which indicated that the CCR of these solutions is higher than 10 °C min^−1^. We conclude that diluted PVS3 solutions from 20% to 60% of PVS3 have very limited vitrification ability but they can be used as a step of graduate sample dehydration [43,54]. Insufficient vitrification ability confirmed previous results when the cell survival after 60%–70% PVS3 treatment ranged from 0 to 0.9% [53].

As in the case of the PVS2-based solutions, two types of Tg were detected in the PVS3-based solutions as well (Figure 2). As a result of water crystallization in diluted PVS3 solutions, the Tg’s of freeze-concentrated solutions were found in a range from −91 to −88 °C (Figure 2). Secondly, the actual Tgs of solutions corresponding to 80 and 100% of PVS3 were detected at −103 and −92 °C, respectively (Figure 2). The Tg of the 100% PVS3 solution measured corresponds to the value of the PVS3 solution already published [38,53,55]. This Tg was close to the Tg’ value of the freeze-concentrated solution detected at −91 °C (Figure 2). We conclude that the PVS3 solute concentration was almost identical to the freeze-concentrated solution (~0.8 g g^−1^) and therefore the PVS3 contains almost no water, which can potentially be frozen at the stable vitrification conditions (6). In this respect, the PVS3 differs from the PVS2. A solute concentration in the original PVS2 (0.65 g g^−1^) is similar to the solution of 80% PVS3 (0.67 g g^−1^) but much less than in the original PVS3 solution (0.78 g g^−1^). A higher concentration of solutes in the PVS3 solution indicates a higher vitrification capacity compared to PVS2, which is in line with previous results [56]. The advantage of the PVS3 solution is less cytotoxicity than in the case of PVS2 so it can be used at room temperature. The PVS3 constraint can be considered rather high viscosity, which can make PVS3 application difficult [57]. On the other hand, the diluted PVS3 solutions, with lower viscosity, in a range from 80 to 100% have acceptable vitrification ability (Figure 2) and have been successfully used as CPAs [42,43,53,54,58,59,60].

### 3.3. Single CPAs

The vitrification ability of the PVS components (a single CPA) had similar characteristics to the combined CPAs tested (PVS2- and PVS3-based solutions). For single CPAs, the acceptable vitrification ability was found in the ranges of solute concentrations 0.5–0.7 g g^−1^ for DMSO, 0.7–0.8 g g^−1^ for EG or Suc, and 0.7–1.0 g g^−1^ for Gly. In these solute concentrations, typically, no water crystallization was found during the C/W cycles. The only 0.7 g g^−1^ Suc solution showed negligible water crystallization (0.6% *w/w*) during the warming cycle. It was close to the consent value (0.2%) for the CCR [52], corresponding to the 80% PVS3 solution (Figure 2). Therefore, we consider the 0.7 g g^−1^ sucrose aqueous solution a threshold value for the Suc vitrification ability. A lower solute concentration than 0.5 g g^−1^ resulted in an unreliable vitrification ability in all single CPAs tested. The vitrification ability was limited by solute crystallization at a concentration higher than 0.9 g g^−1^ in EG and 0.8 g g^−1^ in DMSO, and by the difficult solubility of Suc at a concentration higher than 0.8 g g^−1^. The solute crystallization was described in EG aqueous solution under isothermal conditions [61]. It occurred at high solute concentrations and depended on the time of isothermal conditions. Therefore, we believe that the solute crystallization is not a serious problem of the vitrification method, as the individual components of CPAs cannot be used in a concentrated form due to their toxicity [62]. Glycerol was the only single CPA, which showed the vitrification ability up to 100% concentration.

The two previously identified types of Tg were found in all single CPAs tested. The Tg’ presence was always detected together with the water crystallization during cooling and corresponded to the glass transition of the freeze-concentrated solutions. In diluted Suc solutions, the values for Tg’ and C_g’_ were −46 °C and ~0.8 g g^−1^, respectively. The same values were defined for the Tg’ and Cg’ for the Suc maximal-freeze concentrated solution [29,63]. Therefore, we assume that all values of Tg’ and Cg’ identified in our work correspond, or at least were very close, to the maximally freeze-concentrated solution of the CPAs tested. Secondly, the actual Tg was detected when no or limited water content crystallized during the cooling cycle. Except for two mentioned Tgs, an additional Tg, marked as the Tgs’ was detected as a result of solute crystallization in highly concentrated EG and DMSO solutions (Figure 3 and Figure 4).

An appropriate vitrification ability of single CPAs was always determined by the presence of the Tg together with a limited crystallinity of water or solute (Figure 3, Figure 4, Figure 5 and Figure 6). The lowest solute concentration (0.5 g g^−1^) with the appropriate vitrification ability was detected in the DMSO solution. Our results of the vitrification ability (Figure 3, Figure 4 and Figure 5) correspond to the achievements obtained earlier when the authors defined the glass-forming solute concentrations of 55, 46, and 65% (*w/v*) for EG, DMSO, and Gly, respectively [2]. We suggest the solute concentration 0.7 g g^−1^ as the universal value for obtaining optimal vitrification ability at the CCR and the CWR of 10 °C min^−1^ or higher. The CPAs at the concentration of 0.7 g g^−1^ content provided a small amount of water 0.42 g g^−1^ (water/solute CPAs). Similarly, the low water content of 0.4 g g^−1^ (water/dry mass) was defined as a border value for successful cryopreservation of encapsulated meristems [64]. This cryopreservation method used alginate beads moistened with 0.75 M sucrose, which were air dehydrated above silicagel for 6 h when they reached the final water content of 0.4 g g^−1^ and the corresponding Tg of −67.5 °C. We propose that this Tg value corresponds to the Tg of the concentrated Suc solution. In our study, a similar value of Tg at −67.1 °C was detected in 0.7 g g^−1^ Suc solution with a corresponding water content of 0.42 g g^−1^. Therefore, we suggest the Tg value of −67.5 °C of the air-dehydrated alginate beads [63] was influenced by the Tg of the 0.71 g g^−1^ Suc solution with a corresponding water content of 0.4 g g^−1^. This supports a former finding [65] that the minimal Suc concentration for a glass transition without water crystallization occurred at 70% (*w/w*). We assume that Suc solution acts as a single CPA in the encapsulation–dehydration method due to air dehydration of 0.75 M sucrose to 2.83 M (0.71 g g^−1^). An appropriate osmotic acclimation increases the dehydration tolerance of encapsulated material and allows a decrease in alginate beads’ water content up to 0.36 g g^−1^ [66] and decreases the risk of water crystallization. The high Tg value of Suc solution in comparison with other single cryoprotectants (Figure 3, Figure 4, Figure 5 and Figure 6) should be taken into account when the combined CPAs are designed [2,28]. Including Suc or other saccharides [28] and its proportion in the CPA can influence the Tg of the designed CPA mixture. Accordingly, the Tg of −115 °C in PVS2 with 0.4 M Suc significantly differed from Tg of −92 °C in PVS3 with 1.46 M Suc. The resulted Tg influences the course of sample C/W and the critical storage temperature [10,67].

The applicability of the suggested solute value of 0.7 g g^−1^ is very often limited by solute toxicity, especially in the penetrating CPA [25]. It was proved that the CPA become increasingly toxic as concentration increases [25]. On contrary, a cryopreservation method employing 10% DMSO combined with the rapid C/W rates was successfully used [68]. However, this method is not based on the sample vitrification because the low DMSO concentration did not avoid water freezing (Table 1), so the method was named the droplet-freezing method [69]. The DMSO induces some ultrastructural changes in treated cells that helped to overcome water freezing without fatal damage [70,71] even under nonequilibrium conditions [72]. This can be probably a significant factor that supports including DMSO in the combined CPAs and frequency of the DMSO-based solution used. Currently, more than four times number of papers are available for the PVS2 cryopreservation compared to the PVS3 in the WOS database, reflecting the generally higher efficiency of the PVS2 solution compared to the PVS3. This phenomenon can be explained by a different mechanism of actions of penetrating and non-penetrating CPA [73] related to the specific tolerance of cryopreserved material to CPA toxicity or osmotic stress in the treatments by PVS2 or PVS3, respectively [74]. The higher osmotic effect of PVS3 compared to PVS2 is probably due to the lower penetrability of the CPA component [73] together with the higher solutes concentration in the solution. Sensitivity to osmotic stress or insufficient osmotic acclimation of cryopreserved material can limit the PVS3 use [75]. Recently, some evidence of the harmful effect of DMSO on living organisms or genetic stability has put downward pressure on the proportion of DMSO in the CPA used [76]. 

Regardless of a possible DMSO controversy, we can generally conclude, the low CPA concentrations can be successfully used for the freezing cryopreservation methods, but not the vitrification ones because low concentrations of any single CPA did not show appropriate vitrification ability. A possible solution of the CPA toxicity is a design of the CPA mixture composition with the nontoxic effect of the components [13,25,77]. The crucial factor for the vitrification ability of CPAs is mostly a proportion of water in the solution, which can be up to 30% *w/w* according to our results (Figure 1 and Figure 2), which is in an agreement with the previous findings [65]. On the other hand, based on our results and the results of other authors [2], the penetrating CPAs can be efficient in lower concentrations due to their higher vitrification ability than the non-penetrating CPAs. As we showed, the DMSO proved the best vitrification ability of the CPA tested at a concentration of 0.5 g g^−1^ (Figure 4). Although the 0.7 g g^−1^ solute concentration can be the universal vitrification concentration, the wider region from 0.5 to 0.8 g g^−1^ can conditionally be used concerning the proportion of single CPAs and the corresponding CCR or CWR. 

**Figure 7:**
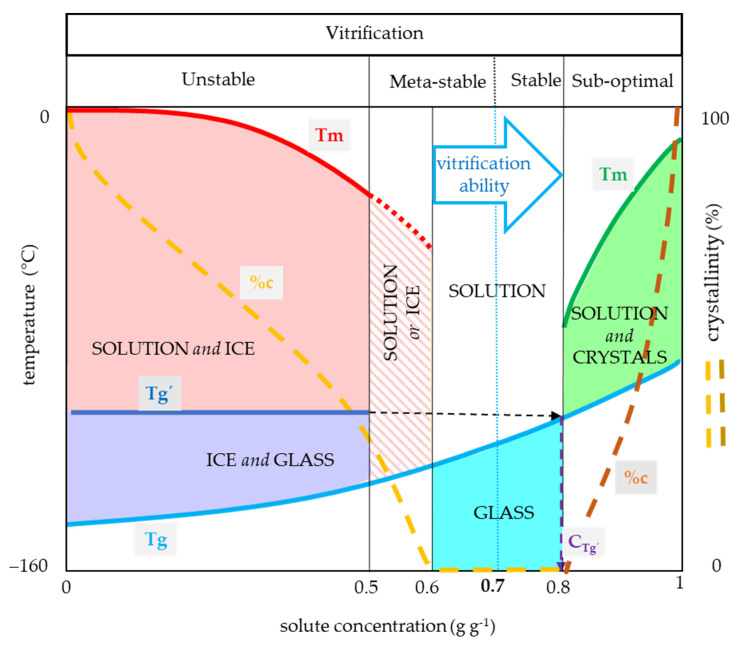
General CPA thermal diagram demonstrating the effect of solute concentration on vitrification ability (VA). Four areas of solute concentration differ in their vitrification ability: (1) low concentration (0–0.5 g g^−1^) results in crystallization of water during cooling with the exception of an extreme CCR, (2) medium concentration (0.5–0.6 g g^−1^) carries the risk of crystallization of water during warming, (3) optimal concentration (0.6–0.8 g g^−1^) for the glass transition, (4) high concentration (0.8–1.0 g g^−1^) carries the risk of solute crystallization, osmotic damage or CPA toxicity. *x*-axis—solute concentration (g g^−1^), left *y*-axis—temperature (°C), right *y*-axis—percentage of crystallinity of water or solute (% *w/w*), Tg—glass transition temperature of the solution, Tg’—glass transition temperature of the freeze-dehydrated solution, Tm—melting point of water (red line) or solute (green line), %c—percentage of crystallinity of water (orange) or solute (brown), C_g_—concentration of the freeze-concentrated solution. The solute concentration of 0.7 g g^−1^ represents the proposed universal vitrification concentration of CPAs.

Based on our results, four typical concentration regions with specific thermal characteristics can be identified (Figure 7) among the CPA concentrations. The first region was characterized by the water crystallization during the cooling cycle and the presence of Tg’ of the freeze-concentrated solution originating from the water crystallization, and it usually ranged from 0 to 0.5 g g^−1^ (Figure 7). This region corresponds well to the unstable vitrification demonstrated earlier on glycerol solution [13]. The second region was typically characterized by water crystallization only during warming and the presence of the Tg corresponding to the actual solute concentration which ranged mostly from 0.5 to 0.6 g g^−1^ (Figure 7). This region corresponds to the metastable vitrification [13]. The third region was characterized by the presence of the actual solution Tg without significant water crystallization and ranged usually from 0.6 to 0.8 g g^−1^ (Figure 7). This region partly belongs to the metastable vitrification conditions [13], which moderately depends on C/W rates, and it ends at the stable vitrification conditions [13], which are independent of C/W rates. The C_g’_ occurred in this range and represented the concentration of the freeze-concentrated phase. It indicated the proximity of the stable vitrification concentration. The fourth region represents sub-optimal conditions and was characterized by the presence of solute crystallization and can occur at a solute concentration higher than 0.8 g g^−1^ (Figure 7).

Moreover, some transient zones can be detected: the simultaneous occurrence of the water crystallization and the actual glass transition in Gly or Suc solutions during the cooling cycle (Figure 5 and Figure 6); the solute crystallization absence in the fully concentrated Gly (Figure 5). Concerning the vitrification ability at the standard C/W rates of 10 °C min^−1^, we recommend the third region of the CPA concentrations (0.6–0.8 g g^−1^) for safe cryopreservation utilizing vitrification methods without any risk of water crystallization at the metastable or stable vitrification conditions with corresponding CCR and CWR. A high solute concentration, close to the C_g’_, is strongly recommended when the C/W rates are limited due to method or sample specificity. The presence of some very effective CPA components as DMSO can shift this region to the lower values. The second region (0.5–0.6 g g^−1^) is applicable in the case of very high C/W rates. The advantage of this region is an opportunity for the CPA concentration decrease to avoid its toxicity. The first region (0–0.5 g g^−1^) is recommended mostly for the freezing cryopreservation method and its use for vitrification is possible only under extreme conditions—very small sample size together with ultrarapid C/W rates. The appropriate CCR and CWR should be verified. The last region of the CPA concentrations (0.8–1 g g^−1^) is inapplicable due to unstable supersaturated solution presence or extreme CPA concentration. 

## 4. Materials and Methods

Osmotic mixtures of two common combined CPAs and their single osmotic components were tested by differential scanning calorimetry. Osmotic components of PVS2 consist of 15% (*w/v*) ethylene glycol, 15% (*w/v*) DMSO, 30% (*w/v*) glycerol, and 13.7% (*w/v*) sucrose [46]. Nutrients—inorganic salts of low concentration according to Murashige and Skoog [78] were omitted; instead, the osmotics were diluted in water. The above-mentioned composition of the original PVS2 solution was defined as 100% PVS2. Next, six solutions were prepared by dilution of the original solution in water to the following concentrations: 90% (*w/v*) PVS2, 80% (*w/v*) PVS2, 70% (*w/v*) PVS2, 60% (*w/v*) PVS2, 50% (*w/v*) PVS2, and 40% (*w/v*) PVS2. 

The second combined CPA, PVS3, consists of 50% (*w/v*) glycerol and 50% (*w/v*) sucrose in water [53]. The original concentration of above mentioned osmotics was defined as 100% PVS3. Next, six solutions were prepared by dilution of the original solution in water to the following concentrations: 80% (*w/v*) PVS3, 60% (*w/v*) PVS3, 40% (*w/v*) PVS3, and 20% (*w/v*) PVS3.

Single components of the above-mentioned complex mixtures were diluted in water to the following concentrations: 0, 10, 20, 30, 40, 50, 60, 70, and 80% (*w/w*) of ethylene glycol (EG), dimethyl sulfoxide (DMSO), glycerol (Gly), and sucrose (Suc). In the case of liquids (EG, DMSO, Gly), next two concentrations were prepared: 90 and 100% (*w/w*).

Thermal analysis was performed by differential scanning calorimetry using TA 2920 (TA Instruments, New Castle, DE, USA) and an LN cooling system (LNCS), using helium as a purge gas and hermetically sealed aluminium pans in three repetitions. Standard C/W rates of 10 °C min^−1^ were used in a temperature range from +25 to −140 °C during the cooling cycle of measurement and a range from −140 to 30 °C during the warming cycle of the measurement. Measured thermal events of prepared CPA solutions were evaluated by the Universal Analysis software (TA Instrument, New Castle, DE, USA). The presence of endotherms (crystallization), exotherms (melting), and the glass transitions during the C/W cycles of the measurement were detected. The temperature of glass transition (Tg), the temperature of onset melting peak (Tm) was measured. The amount of crystallinity was calculated based on the measured heat of fusion. Standard heat of fusion of 334, 181, and 173 J g^−1^ K^−1^ was used for water, EG, and DMSO, respectively.

## 5. Conclusions

The appropriate vitrification ability of two combined (PVS2 and PVS3) and four single (EG, DMSO, Gly, Suc) CPAs usually ranged from solute concentrations of 0.6 to 0.8 g g^−1^ for the CCR and the CWR of 10 °C min^−1^ or higher. Gly was the only single cryoprotectant which possessed vitrification ability up to 100% solute concentration. DMSO was the most effective CPA with an appropriate vitrification ability already starting at a solute concentration of 0.5 g g^−1^. We demonstrated that the Suc solution can be used as a natural single CPA when dehydrated to a concentration of 0.7 g g^−1^ or higher. In the combined CPAs, the appropriate solute concentration was proved only in 100% PVS2 or 80 and 100% PVS3. 

No crystallization in all CPAs during the cooling cycle is associated with the detection of the Tg corresponding to the actual solution concentration. The presence of a glassy state of the freeze-concentrated solution, characterized by Tg’, indicates water crystallization occurrence during the cooling cycle and a possible injury of a sample by ice crystal growth. On the other hand, the Tg’ value can help to identify the C_g’_ of the maximally freeze-concentrated solution, which corresponds to the solute concentration typical for the stable vitrification conditions [13]. The solute concentration of 100% PVS3 close to the C_g’_ suggests its higher vitrification ability even at very low CCR and CWR compared to 100% PVS2.

Generally, combined CPAs are recommended to avoid the risk of sample damage due to CPA cytotoxicity [25,79]. The design of combined CPAs can decrease the portion of the individual components below their toxicity threshold [79]. While the higher proportion of DMSO in the combined CPA decreases the total solute concentration with appropriate vitrification ability, the higher proportion of Suc increases the Tg value of the CPA mixture and subsequently increases the safe storage temperature of samples. Knowledge of the CPAs thermal properties can help to design a new combined CPA with the appropriate vitrification ability.

## Figures and Tables

**Table 1:** Characteristics of cryopreservation methods with respect to acclimation and dehydration.

Acclimation ^1^	Pre-Treatment ^2^	Extensive Dehydration ^3^	Cooling Rate ^4^	Cryopreservation Method ^5^
Cold	None	Freezing	Slow	Two-step/slow-cooling/controlled-freezing
	Diluted CPAs	Freezing	Rapid	Droplet-freezing
	Loading solution	CPAs	Rapid	Vitrification
Osmotic	Osmotic solution	Air-dehydration	Slow	Encapsulation–dehydration
	Osmotic solution	Air-dehydration	Rapid	Encapsulation–dehydration
	Osmotic solution	CPAs	Rapid	Encapsulation–vitrification
	Loading solution	CPAs	Rapid	Vitrification
None	Loading solution	CPAs	Rapid	Vitrification
	Diluted CPAs	Freezing	Slow	Two-step/slow-cooling/controlled-freezing
	Diluted CPAs	Freezing	Rapid	Droplet-freezing

^1^ Acclimation means the long-term action of low temperature and/or moderate osmotic stress. ^2^ Pre-treatment is the exposition of isolated shoot tips/meristems/cells to a diluted solution of osmotically active components: the osmotic solution is a common designation of diluted osmotic solutions, usually saccharides, the loading solution is a specific type of the osmotic solution, which usually consists of glycerol and sucrose. ^3^ Extensive dehydration is performed by either: extracellular freezing, air-dehydration above silicagel or in a laminar flow-bench, or CPAs (Cryoprotective Agents) representing a mixture of highly concentrated penetrating and nonpenetrating cryoprotectants. ^4^ Cooling rate influences the final state of matter: a slow cooling rate (degrees per hour) provides controlled (equilibrium) freezing conditions resulting in ice crystals production, a rapid cooling rate (usually thousands or hundreds of degrees per minutes) represents (nonequilibrium) vitrification conditions avoiding water crystallization. ^5^ Cryopreservation methods list general designations of cryopreservation protocols [3,7,10].

## Data Availability

The data presented in this study are available on request from the corresponding author.
